# A latent approach in the fashion retailing context: segmenting co-creator users

**DOI:** 10.1186/s40691-022-00323-7

**Published:** 2023-03-25

**Authors:** Carlota Lorenzo-Romero, María-del-Carmen Alarcón-del-Amo, Marta Frasquet-Deltoro

**Affiliations:** 1 Department of Business Administration, Faculty of Economics and Business, University of Castilla-La Mancha, Plaza de La Universidad, 1. 02071, Albacete, Spain; 2 Department of Marketing, University of Murcia, Murcia, Spain; 3 Department of Marketing, University of Valencia, Valencia, Spain

**Keywords:** Fashion retailing sector, Co-creation values and activities, Engagement, Satisfaction, Spanish users’ latent profile

## Abstract

Co-creation activities have shown dramatic development somewhat recently. The initial step of a productive co-creation technique originates from a comprehension of people’s practices inside of these websites. Based on the limited knowledge of online co-creation behaviour in the retail sector, particularly in fashion retailing, this study develops a latent class segmentation analysis that allows examining the diversity of co-creation behaviours. Thus, the main objective of this paper is to analyse the heterogeneity of co-creators’ behaviours in the online retail environment. This study examines different indicators that determine the co-creation behaviour of users such as co-creation value and activities, engagement with the company/brand, satisfaction with the co-creation process, intention to continue co-creating) in the Spanish fashion sector. Three different segments have been obtained: “full co-creator”, “co-creator oriented to the company/brand” and “co-creator oriented to other people” that show three distinct ways of co-creating with a fashion firm. The findings offer fashion retailers an interesting perspective for attracting customers to co-creation ventures during the fashion online shopping process.

## Introduction

New innovations are a challenge for all areas, causing advertisers to reconsider the most suitable approaches with which to attract an inexorably youthful public who are considerably more familiar with the utilisation of digital gadgets to collaborate with brands. Although a few groups actually need to see and feel items at global catwalks and fairs (Hägg & Preiholt, 2006), the advanced age offers prospects that are starting to impact the promotion of extravagant products and, along these lines, the patterns of advertising systems in a mass market style.

The fashion sector, understood to encompass “clothing, accessories, footwear, jewellery, watches, and cosmetics” both industrially and commercially (Lorenzana, 2018), represents around 2–4% of the global gross domestic product (GDP) (Fashion United, 2020) and employs around 300 million workers worldwide (Modaes, 2018). In Spain, this sector represented and maintained its weight in the Spanish economy at 2.8% of the GDP in 2019, the year prior to the beginning of the COVID-19 pandemic (Corner, 2020; Forbes, 2020; Modaes, 2020a), accounting for 4.1% of active jobs in the labour market (EY, 2020; Lorenzana, 2018) and 8% of the industrial sector (Lorenzana, 2018).

The dynamism of the Spanish economy thanks to this sector is due to the entire production and distribution chain being organised around this industry, as it includes companies dedicated to design and manufacturing, as well as those dedicated to distribution, logistics and intermediaries through 20,000 industrial companies and 60,000 points of sale, thus representing 10% of Spain’s productive fabric. It should be noted that this sector attracts 13% of global shopping tourism and represents 8.7% of exports (EY, 2019). In addition, it is worth noting that since 2016, purchases in physical shops have increased from 40 to 48% and through digital channels from 19 to 30% (PwC, 2019).

The fashion sector is one of the main online shopping sectors in Spain, behind only tourism and air transport. The sector’s weight in the total volume of e-commerce sales in Spain is growing exponentially. In 2012, its volume barely exceeded 1% of the total, i.e. one out of every 100 items of clothing, footwear or accessories was bought online, but by 2019 it had almost reached 7% (Modaes, 2020b). Even in dark times for the fashion industry, Spanish companies in the sector found a revulsive to boost their business: during the year 2020, the sector gained 2126 exportation companies in comparison to 2019, with a total of 22,896 companies with activity in international markets (Modaes, 2021).

The speed with which garments circulate in the market together with the style of consumption, based on the satisfaction of buying quantity at an affordable price and with attractive designs, is what would make up so-called fast fashion. However, as consumers are becoming more informed and demanding, motivated by applying their social interests to their purchasing choices (Harrison, 2005), and aware of the well-being of society (Martínez-Cañas et al., 2016) and their own emotional well-being (Jägel et al., 2012), other trends have emerged within the fashion sector. In contrast to fast fashion, “slow fashion” has emerged, characterised by an appreciation for better quality and, therefore, longer-lasting garments and fabrics, thus putting the brakes on the mass consumption of clothes identified with “fast fashion”. Consumers interested in ‘slow fashion¡ “support artisanal products, promote smaller, local fashion companies or those close to the consumer and are committed to ecological and sustainable materials”.

The evolution of the Internet and new technologies has propelled a change in business practices and, in particular, this context has proven to be very opportune for the dissemination of “co-creation” activities, wherein a customer actively collaborates with a company to create value (Chepurna & Criado, 2021). For example, IKEA launched in 2018 a digital platform named “Co-Create Ikea” to encourage customers to develop new offerings”. Furthermore, López-Navarro and Lozano-Gómez (2014) believe that co-creation is “an appropriate strategy to achieve sustainability (p.6), since by integrating customers into the value creation chain, garments are created that can better and longer satisfy basic human needs and also create an “emotional bond” (p.7) with those garments.

Digital marketing strategies in fashion retailing, through communication by means of social networks, websites, bloggers, or influencers, are essential to innovation in the ways of interacting with consumers, and in the design of communication and distribution systems. Brands need to reinvent their strategies to adjust to and approach developing business sectors of consumers who are eager to consume fashion (González-Romo & Plaza-Romero, 2017), generating customised responses to users to create motivational experiences during their online shopping process (Lorenzo-Romero & Constantinides, 2019). Firms are developing customer co-creation activities because they contribute to firms’ outcomes, as they help to establish more grounded connections and increase commitment (Prahalad & Ramaswamy, 2004a).

Despite the opportunities that online co-creation activities offer to firms, previous researchers have paid more attention to developing conceptual papers (e.g. Ramaswamy & Ozcan, 2016; Zwass, 2010). Among those empirical papers, the ones referring to retail co-creation experience are very scarce (e.g. Anaza, 2014; Frasquet-Deltoro et al., 2019; Gebauer et al., 2013; Thomas et al., 2020). In addition, co-creation includes different activities (co-promotion, co-creation of branding, co-design, co-production of products, and so on), but literature on how fashion brands can implement co-creation is still limited (Thomas et al., 2020). Additionally, most papers study co-creation activities from a platform design perspective (Kang, 2014, 2017) and investigate how to motivate consumers’ participation, but do not specify the co-creation activities (Thomas et al., 2020). Moreover, the limited literature in this field has not investigated the heterogeneity among online co-creators through a segmentation approach. A segmentation study is useful for both fashion retailers and consumers, as it allows offering customised products that increase engagement and satisfaction with a brand. In this research, we suggest a classification of co-creator users, utilising a wide range of behavioural and sociodemographic variables. To achieve this objective, we apply a latent class segmentation approach, which (to our knowledge) has not been previously used in the context of online fashion co-creation, allowing a more reliable and exact segmentation process.

Therefore, the main research objective is to analyse the heterogeneity of online co-creators. This main objective can be divided into the following specific research objectives:

1. To analyse the co-creation value (CV) experienced by users when they are actively participating with a specific fashion company/brand.
2. To study the different co-creation activities (CA) that users develop during their co-creation process with a specific company/brand.
3. To obtain a latent segmentation model that allows identifying segments of online co-creators based on co-creation value, co-creation activities, satisfaction, engagement, and intentions to continue co-creating, and to suggest marketing strategies to practitioners for different customer profiles.

## Literature review

### Value creation strategies in the digital fashion environment

In light of multiple advantages that online channels offer to companies within the fashion industry, companies focus on the joint creation of personalised experiences as an appropriate strategy for meeting basic human needs (López-Navarro & Lozano-Gómez, 2014). This strategy provides unique experiences and creates garments with emotional meaning, resulting in an attachment not only to the garments, which are retained for longer, but also to the brand values, initiating a lasting and profitable relationship between brands and customers (Park et al., 2010).

The term ‘co-creation’, coined by Prahalad and Ramaswamy (2004b), refers to the process of companies developing products, services and experiences jointly with their stakeholders, opening up a new world of value. Customers, as an essential group of stakeholders, and the company are closely committed to creating truly unique and rewarding value propositions (Frow & Payme, 2011). This new mindset involves a shift away from the business-centric view of value creation in the fashion industry that views consumers as mere passive actors of a value proposition of trends unilaterally imposed by fashion firms, to a new mindset of human experience that actively involves customers in defining and delivering enhanced value, resulting in enriched and meaningful experiences. This approach aims to break the cycle of passive purchasing behaviour by incorporating consumers into the value creation chain to design garments that build emotional links with firms (López-Navarro & Lozano-Gómez, 2014).

Co-creation cannot take place if a consumer’s value creation process is inaccessible to a firm. Since interaction is a bidirectional process (Ballantyne, 2004; Ballantyne & Varey, 2006), the consumer and the company must engage in activities together, in which both parties are active (Grönroos & Ravald, 2011). That is to say, there must be a willingness by both to participate (Prahalad & Ramaswamy, 2004b). Authors such as Bowen (1986) and Mills et al. (1983) argued as early as the 1980s that if customers correctly express their expectations and companies seek a way in which to meet them, there can be co-creation of value.

Thus, apart from the spheres already existing in the traditional value creation process, which remain separate, with co-creation a new joint sphere is created, wherein the exchange of ideas takes place. For example, when a customer wants to change a product feature and makes suggestions, he or she is influencing a company’s production process. Therefore, according to Grönroos and Voima (2013), it is possible to divide value creation into different spheres:

1. Supplier sphere. A company generates potential value that will later be exploited by a consumer; in other words, in this sphere of value creation the company is a resource provider or, as Grönroos (2011) calls it, a value enabler. However, co-creation does not take place in this sphere, as there is no collaboration.
2. Customer sphere. For Grönroos (2008, 2011), who maintains that the real creator of value is the customer, there is still value creation in this sphere, albeit independently, without contact with the company. In this form of value creation, the customer only interacts with the resources obtained from the company but not with its process (Grönroos & Voima, 2013). Value creation is considered to take place because it combines those resources offered in the market to create value outside of the productive sphere, but co-creation does not take place.
3. Joint sphere. This is where co-creation does take place, since it is where both a company and a customer collaborate to create value, both of whom assume the role of a co-creator. The extent of this sphere will depend on the different tasks performed by a consumer.

In this sense, the adoption of online technologies by firms (Verhoef et al., 2015) allows increasing customer empowerment, while at the same time enabling co-creation in different contexts (Zhang et al., 2017). Customer co-creation behaviours can be performed offline and online, but social media helps to develop online in an easier way (Wu et al., 2017).

The co-creation process can be developed when a customer is forced to participate or is given the option to co-create (Bendapudi & Leone, 2003). Within the second option (voluntary behaviour), several authors have identified two different behaviours: customer participation and customer citizenship (Groth, 2005; Yi & Gong, 2013), which have been demonstrated in the fashion industry (Frasquet-Deltoro et al., 2019). User participation behaviours are predicted actions which can be vital for a carrier to be introduced or for co-creation to be completed, while client citizenship behaviours are richer conducts which can be discretionary and exceed undertaking performance, as a consequence supplying splendid cost for companies (Groth, 2005). Yi and Gong (2013) developed a scale to measure both concepts, suggesting four dimensions: information seeking, information sharing, responsible behaviour, and personal interaction, to capture customer participation behaviour, and other four dimensions: feedback, advocacy, helping, and tolerance to measure customer citizenship behaviour.

Co-creative fashion firms aim to foster emotional ties between customers, brands and garments through the development of positive and meaningful experiences. Thus, within the strategy of experience co-creation, value creation does not only derive from a company’s internal activities (Ramaswamy, 2011). Rather, it is the result of interactions created throughout the system, involving not only products, services and business processes, but also a network of customer communities and a company’s enhanced network, whether internal or external, which, instead of companies and their employees, constitute the new base of value-creating competencies. Therefore, firms should focus on maximising the individual human experiences of customers at all of these key points of interaction that are critical to value creation because, ultimately, the value perceived by customers comes from the sum total of these unique value experiences (Prahalad & Ramaswamy, 2004b). To provide these personalised experiences, fashion firms must design multiple points for possible customer interaction, generating satisfaction in the co-creation process that causes the need of continuing to co-create (Frasquet-Deltoro et al., 2019; Navarré et al., 2010).

### Co-creation activities and behavioural responses after co-creation experiences: engagement, satisfaction, and intention to continue co-creating

Involved shoppers are more likely to express themselves when recommending companies’ products or brands and, therefore, become active ambassadors for fashion firms. In light of the above, it seems to be clear that managers must pay attention not only to the quality of companies’ products, services and processes, but also to the quality of infrastructure for interactions (the experience environment) between firms and consumers, which contribute to the quality of the co-creation experience process (Prahalad & Ramaswamy, 2004a).

Co-creation activities involve communication and interaction with the brand’s staff to create value, typically involving joint participation of other customers. (Nysveen & Pedersen, 2014). Participation platforms are the cornerstone of the co-creation process, as they are a means of creating value between companies and shoppers, which supports the other three components of co-creation: the mindset of experience, the context of interactions, and network relationships (Prahalad & Ramaswamy, 2004a; Ramaswamy & Gouillart, 2010).

This requires going past business cycles to develop engagement experience platforms that support customer participation and enable large-scale, bilateral interactions between businesses and their customers in the ongoing development of personalised co-creation experiences that generate truly unique and valuable value for each customer. Therefore, companies should create the most appropriate and diverse engagement platforms through which business and customer processes come together to create a positive personalised consumer experience with each interaction (Ramaswamy & Gouillart, 2010).

Given the importance of the experience of co-creation with respect to subsequent behavioural responses between an individual co-creator and an organisation, it is necessary to equally identify the relationship between the perceived experience of co-creation and the participation in new actions of co-creation. Satisfaction with regard to an individual’s repurchase intention or repetitive behaviour has been studied in the literature from the perspective that satisfaction improves repurchase intention when consumers are characterised as individuals with a greater preference for risk (Wu & Chang, 2007). Satisfaction with a product or service is therefore critical in determining an individual’s repurchase or reuse intention (Liang et al., 2018).

In addition, when comparing an experience with an individual’s expectations, there is a blend of perspectives, like sentiments and advantages. Positive satisfaction brings about rewarding experiences and, thus, more memorable ones. Furthermore, perceptions of the quality of a service or the satisfaction with the value are also influential with respect to the loyalty and the later behaviours of an individual (Bigné et al., 2001), making it necessary that organisations support high levels of satisfaction with clients to support their continuous relation with them.

In order for this relationship to be extended over time, it is necessary for a series of events to take place. Firstly, communication between the parties must be good and fluid and the supplier must offer a quality service in accordance with what has been established, all of which will be favourable for achieving an atmosphere of trust, i.e. a relationship of interest is established between quality, trust and loyalty, which is the result of the evaluation that a client carries out after the experience (Santoso & Erdaka, 2015). Related to the previous idea, the expectation to continue co-creating is centred chiefly upon the parts of commitment or satisfaction; in the same way, the experience of co-creation exerts a direct and positive influence on personal variables such as satisfaction (Mathis et al., 2016), so if a greater degree of satisfaction with the co-creation is also detected in a shopper, the option of repetition of the co-creating action would have greater possibility of being carried out.

In sum, Anderson and Srinivasan (2003) argue that an individual will participate in the future in co-creation activities if his or her previous experience was satisfactory. By participating in an online co-creation process, customers feel empowered and, as a result, are more encouraged to co-create in the future with an organisation (Füller et al., 2011).

### Segmentation in the fashion sector

Division can improve showcasing adequacy and create or maintain an association’s capacity to profit via recognisable promotional openings (Weinstein, 1987). This asset-based way in which to deal with overseeing associations proposes that division can assist organisations with assigning monetary and different assets all the more viably (McDonald & Dunbar, 2004). Furthermore, division prompts a superior comprehension of customers, which can help in planning more suitable showcasing programmes (Dibb et al., 2002). In this sense, segmentation presents several advantages for a company to improve marketing effectiveness and identify marketing opportunities (Weinstein, 1987). Moreover, it allows a superior comprehension of customers, which can help to design more appropriate marketing strategies (Dibb et al., 2002). This resource-based approach suggests that companies can use this segmentation to assign their resources more effectively (McDonald & Dunbar, 2004).

Quinn et al. (2007), based on Wedel and Kamakura (2000), point out that a manager’s perspective determines how homogeneous groups of potential customers should be identified via market research. This broader approach does not only reveal key benefits for professionals who manage complexity within a dynamic context.

According to Agrawal and Rahman (2015), customers are the most important stakeholders in value co-creation. The roles of customers vary depending on their work in the creation and delivery of the offer, and Mills et al. (1983) and Bowen (1986) argue that if customers know their role in the process, they can be integrated into the value co-creation activity. Depending on the sphere of consumer influence, consumers can assume different roles; Agrawal and Rahman (2015) classified the roles of consumers based in value co-creation as follows:

1. Consumer as a “co-producer”. Lengnick-Hall et al. (2000) define co-production as a process in which customers participate in the work of a firm. When a consumer acts as a co-producer, he or she is involved in the production process almost from the beginning. This term encompasses all activities that are characteristic of the production phase. A consumer who is a co-producer is also a co-designer, co-manufacturer or co-promoter, for example.
2. Consumer as a “co-distributor”. If the distribution function is responsible for bringing final goods and services to a consumer, the consumer is a co-distributor when he or she collaborates on this task (e.g. IKEA).
3. Consumer as a “co-promoter”. The rise of social media has given more power to customers and allowed them to share their experiences with brands. Companies have decided to take steps to engage their customers, cultivate relationships, and prevent negative word of mouth (Zwass, 2010).
4. Consumer as a “co-manufacturer”. Consumers can also contribute their knowledge and skills by producing content for a company engaged in, for example, social media, such as YouTube and Instagram. These companies are maintained thanks to the posts and videos created by the users themselves, who, on the one hand, consume these social networks and, on the other hand, are content creators, being at the same time both consumers and manufacturers.
5. Consumer as a “creator of experiences”. Woodruff (1997, p.142) argues that “value comes from preferences or evaluations learned by consumers and not only from market supply”. It is interesting that customers are part of the innovation process in the consumer experience, as they provide suggestions based on experiences already lived (Castelló, 2015) regarding all of the elements that are decisive in the customer journey, from the pre-sales process to the technical assistance phase, and, in addition, help to achieve satisfaction and loyalty (Rahman, 2006).
6. Consumer as a “co-ideator”. A customer acts as a co-ideator when he or she contributes ideas to a company in some way, e.g. through comments on a social network or on a platform that the company has made available for this purpose. Co-creation allows customers to contribute innovation and creativity (Sánchez-González & Herrera, 2014) in the creation of value in the market. In addition, the exchange of ideas between companies and customers can encourage the participation of other customers (Agrawal & Rahman, 2015). The concept of the co-ideator reaches far beyond mere product design, as it can provide ideas for improving customer support or production technology, for example.
7. Consumer as a “co-evaluator”. Ideas brought to a firm, whether from the firm or from consumers, can be evaluated by customers in order to know more about the likelihood of their acceptance and the consequences of their development. However, Agrawal and Rahman (2015) caution that this tool should be used with great care because it could, in the event that the product is not accepted by the co-evaluators, lead to negative publicity or bring a bad reputation to a brand if done openly and publicly.
8. Consumer as a “co-designer”. When consumers participate in the design process of a good or service, they are acting as “co-designers” (e.g. H&M).
9. Consumer as a “co-teaser”. Companies can benefit from involving customers in the testing of a product before it is launched in the market, as this allows them to check the reactions to and evaluations of the product in advance and, if necessary, rectify its features, thus increasing the likelihood of its success.

The fashion retail sector provides a relevant context here since the heterogeneity of customers in this sector makes it necessary to segment them to offer more adapted strategies. By their very character, fashion markets are characterized with the aid of using volatile demand, and elements like age, income, and way of life appear to affect a selected and regularly divided marketplace environment (Hines & Bruce, 2001).

In terms of age, Salesupply (2019), Modaes (2020b) and ISDIGITAL.LAB (2016) agree that those who buy the most fashion online are between 31 and 45 years of age. This is in line with the age group that buys the most online for any type of product, not only fashion (IAB Spain, 2020). Modaes (2020b) widens the range: 49% of all online fashion shoppers are aged 35–54. The younger population (39%) is divided between 22% of 25–34-year-olds and 17% of 16–24-year-olds. Furthermore, it states that older generations are becoming increasingly relevant in the digital marketplace, with 12% of total online fashion shoppers aged 55–74. Distinguishing according to their level of education, online fashion shoppers with secondary education stand out (43%). Although closely followed by those with higher education (35%), fashion consumers with lower levels of education increase their share year after year (Modaes, 2020b). According to their level of income, 28% of online fashion shoppers have an income of more than 2500 euros per month, almost the same as those with incomes of 901–1600 euros (25%) and 1601–2500 euros (23%). The group with a lower income of less than 900 euros per month are those who buy fashion online the least, i.e. only 8%, although they have also been increasing their share over the last few years (Modaes, 2020b).

ISDIGITAL.LAB (2016) differentiates between “heavy digital buyers”, who shop online more than once per month, “medium digital buyers”, who shop every one or two months, and “light digital buyers”, who shop once per quarter or less. Most online fashion shoppers tend to fall into the latter category, with “heavy digital buyers” being the least frequent. On the other hand, the percentage of “heavy digital buyers” is higher among men (15%) than among women (8%), although the proportion of “medium digital buyers” is almost identical and, therefore, the remaining quota of “light digital buyers” is higher among women than among men. However, there are hardly any notable differences in terms of age between these categories, although “heavy digital buyers” are particularly concentrated in the 25–44 age range, i.e. those belonging to “Generation X” and “Millennials”. It is noteworthy that the youngest, “Generation Z”, occupy a higher proportion of consumers with medium purchase intensity (“medium digital buyers”) in comparison to the other two types. Analysing the geographical locations of “heavy digital buyers”, the habitual buyer of fashion on the Internet is located in the centre of the peninsula, especially Castilla-La Mancha, Castilla y León, and Extremadura, in addition to the Levantine area, but is in the minority in Madrid and Barcelona, probably due to the greater supply in physical establishments that can be found in large cities (ISDIGITAL.LAB, 2016).

On the other hand, as for the motivations that lead consumers to make online purchases, the main ones are price and convenience, with 27% stating that they find better prices online than in physical shops, and 22% stating that they find it more convenient to buy online because they avoid going to a physical shop. Likewise, 16% stated that they find better deals, 13% stated that they find more availability of items or sizes, and 11% stated that they value the fact that there are no closing hours. Only 2% stated that they buy online based on the opinions of other shoppers. However, if we break down these totals by gender, we arrive at the conclusion that men attach greater importance to the “price” factor and women do so to the “convenience” factor.

Very frequently, retailers focus on a specific segment of customers (Bevan, 2001; McGoldrick, 2002). Neverthless, capability clients are not constantly smooth to identify, that is evidenced via way of means of perennial discounts and unsold stock. In general, most companies begin by proactively designing interactive platforms with small groups of chosen people somewhere in the system, for which they segment and focus on the most proactive consumers or influencers. This platform design then evolves as a function of the co-creative process, extending the scope and scale of interactions by incorporating a larger number of players and other linked platforms, thus expanding the space and possibilities for generating valuable experiences.

In light of the aforementioned literature, we propose in this paper the generation of a segmentation study with which to analyse co-creator user profiles based on their experiences of value and co-creation activities with a given fashion brand, depending on the link that they have with it, their satisfaction with those experiences, and their behavioural response to the intention to co-create in the future. Therefore, we pretend to answer the following research questions (RQ):

RQ1: Are co-creator consumers homogenous based on co-creation value, co-creation activities, and behavioural responses? Is there heterogeneity?

RQ2: Are co-creation value, activities, engagement, satisfaction, and intention to co-create in the future variables that are useful to segment co-creator users?

RQ3: Are there sociodemographic differences between segments?

## Methods

This study focuses on the fashion retail sector in the context of Spain. According to data from the National Commission for Markets and Competition, the Spanish fashion sector recorded a turnover of 1062 million euros in the first half of 2018, i.e. a 30% increase year on year (Retail, 2019). In 2018, 21.6 million Spanish people bought something on the Internet (3.9 million new online buyers) and more than 13.7 million purchased clothing, footwear and accessories (iTrend, 2019). In fact, the multinational Inditex stated that 10% of its total profits came from e-commerce. It does not seem that this growth in online sales within the fashion sector will stop in the near future, nor does it seem that there is a limit to such growth and if there is, it is set by the consumers. In fact, experts estimate that in a couple of years the online turnover of some brands could reach between 30 and 40% (Digital, 2019). This situation places the sector in third place among all industries in terms of turnover in e-commerce, representing 6% of all e-commerce in Spain, and with 5.4% of total sales in fashion (Moda.es, 2019).

This research was developed using an online questionnaire aimed at active online co-creators in the fashion retail sector in the context of Spain. The survey was sent to an online panel of a market research company, specifically to customers with experience in co-creation with retailers (the definition of co-creation was explained in detail so that the participants were clear about the term). Respondents had first to identify the retailer they had most recently co-created with and answer to the subsequent questions with that specific retailer in mind. An extensive list of retailers’ names was provided; alternatively, respondents could write a valid name. The final sample size was 400 individuals.

The measures for the variables used in our study were mainly scales validated in previous online co-creation studies. For co-creation value, a multidimensional scale was used that was taken from Yi and Gong (2013) (see Table 1), and all items were measured using seven-point Likert scales (1 = completely disagree, 7 = completely agree). For co-creation activities, a scale was created based on the one used by Constantinides et al. (2015) and Nysveen and Pedersen (2014) (see Table 2), and they were measured using a seven-point frequency scale ranging from “Never” (1) to “Every day” (7). Moreover, the behavioural response variables (engagement, satisfaction, and intention to continue co-creating) were measured using seven-point Likert scales (see Table 3).

**Table 1:** Co-creation value scale

Factor		Item
Information seeking	CV1	I asked others for information on what *the company/brand* sold
	CV2	I searched for information on where to get products/services of *the company/brand*
	CV3	I paid attention to how others use the products/services in *the company/brand*
Information sharing	CV4	I clearly explained to *the company/brand* what I wanted to do
	CV5	I gave *the company/brand* proper information
	CV6	I provided necessary information so that *the company/brand* could perform its duties
	CV7	I answered all of *the company/brand’s* product/service-related questions
Responsible behaviour	CV8	I performed all of the tasks that *the company/brand* required of me during the co-creation process
	CV9	I adequately completed all of the expected behaviours when I participated in the co-creation with *the company/brand*
	CV10	I fulfilled responsibilities to *the company/brand* when I co-created
	CV11	I followed *the company/brand’s* directives or orders when I participated
Personal interaction	CV12	I was friendly to *the company/brand* or the person who attended to me when I participated in the co-creation
	CV13	I was kind to *the company/brand* or the person who helped me during participation
	CV14	I was courteous to *the company/brand* or the person who helped me during participation
	CV15	I was polite to *the company/brand* or the person who helped me during participation
	CV16	I didn’t act rudely with *the company/brand* or the person who helped me during participation
Feedback	CV17	If I had a useful idea on how to improve a product/service, I let *the company/brand* know
	CV18	If I received a good product/service from *the company/brand*, I commented on it
	CV19	If I experienced a problem, I let *the company/brand* know about it
Advocacy		After my interaction with *the company/brand* …,
	CV20	I said positive things about *the company/brand* and its product/service to others
	CV21	I recommended *the company/brand* to others
	CV22	I encouraged friends and relatives to participate with *the company/brand*
Helping	CV23	I assisted other consumers if they needed my help on issues related to *the company/brand*
	CV24	I helped other consumers if they seemed to have problems in aspects related to *the company/brand*
	CV25	I taught other consumers to correctly use the product/service offered by *the company/brand*
	CV26	I gave advice to other consumers on issues related to *the company/brand*
Tolerance		In the case that you finally bought the product after your co-creation experience …,
	CV27	If the product/service was not delivered as expected, I would be willing to put up with it
	CV28	If *the company/brand* made a mistake during product/service delivery, I would be willing to be patient
	CV29	If I had to wait longer than normally expected to receive the product/service from *the company/brand*, I would be willing to adapt

Words in italics are those that in the online questionnaire were changed as per the chosen retailer with whom they co-created most recently

Source: Yi and Gong (2013)

**Table 2:** Co-creation activities scale

	Item
CA1	I share my opinions in fashion web spaces (email, website of the retailer, social network of the brand, price comparison engine, forum, blog, etc.)
CA2	I discuss with other online users aspects of a garment or accessory (design, price, usage)
CA3	I provide ideas on the Internet about how fashion companies can create and/or improve their products and services
CA4	I participate in decisions about the development of products and services offered by fashion companies
CA5	I find solutions to my problems together with the company
CA6	I am actively involved in the development of new products and services in the fashion industry (e.g. buying a product that you have previously customised)
CA7	I participate in online polls suggested by a fashion company to market the most voted product and/or disseminate the most voted spot
CA8	I give my opinion, over the Internet, about beta tests (tests of products that have not yet been launched in the market) of garments and accessories
CA9	I participate, via the Internet, in the financing of products that I find interesting to bring to the market

Source: Adapted from Nysveen and Pedersen (2014) and Constantinides et al. (2015)

**Table 3:** Behavioural responses: engagement, satisfaction, and intention to continue co-creating

Scale		Item	Source
Engagement	E1	Co-creating with *the company/brand* allowed me to feel valued	Adapted from Blasco-Arcas et al. (2014), Medlin and Green (2003) and Sprott et al. (2009)
	E2	*The company/brand* interested me by suggesting co-creation activities	
	E3	The experience provided me a special interaction with *the company/brand*	
	E4	I felt identified with *the company/brand* by the fact that they allowed me to participate actively	
	E5	My image of *the company/brand* has improved from the experience	
Satisfaction	S1	I think that I made the right decision performing this kind of activity	Adapted from Navarré et al. (2010) and Oliver (1980)
	S2	The co-creation experience with *the company/brand* was successful	
	S3	I am satisfied with the activity of online co-creation offered by *the company/brand*	
	S4	I am satisfied with the way *the company/brand* managed the online co-creation activity	
Intention to continue co-creating	IC1	Given the opportunity, I would like to collaborate with *the company/brand* in generating ideas for new products/services in the future	Adapted from Blasco-Arcas et al. (2014)
	IC2	I would like to participate in defining the products/services that I would buy from *the company/brand*	
	IC3	Given the opportunity, I would like to take an active part in any act of co-creation offered by *the company/brand*	

Words in italics are those that in the online questionnaire were changed as per the chosen retailer with whom they co-created most recently

The first step is the purification of items, testing the validity and reliability of the scales. Therefore, the transparency of the observed variables was performed through exploratory factor analysis (EFA) through SPSS. Factor construction was conducted in EFA by means of principal component analysis (PCA). After conducting EFA, confirmatory factor analysis (CFA) was performed through a structural equation modelling technique by means of EQS software for the verification of recognised factors. To develop these factor analyses, we have chosen to analyse the scales separately, since, according to Groth (2005) and Yi and Gong (2013), co-creation value is a second-order construct composed by customer participation behaviour (CPB) and customer citizenship behaviour (CCB); therefore, we should proceed in two steps to develop the factorials as suggested by Ulaga and Eggert (2005). Thereafter, the resulting factor scores of the measurement model were used as a measure of these indicators, so these variables are continuous, as defined in the real intervals (Allred et al., 2006; Brown et al., 2003; Mäenpää, 2006). The main objective of this research is to analyse the existence of heterogeneity among co-creator consumers, if found, to group them into different segments that are as homogeneous as possible and more heterogeneous regarding the remaining groups. To achieve this aim, a latent segmentation methodology through Latent Gold 4.5® statistical software was used to define the segments and profile the individuals. The factor scores created during this process were used as variables (indicators) to develop a cluster analysis based on the variables measured as continuous variables. The exogenous variables used to describe the clusters, also known as covariates (Vermunt & Magidson, 2005), were gender, age, level of education, work situation, marital status, and income as covariates to profile co-creators in the fashion retail sector. In addition, the interest in using latent class segmentation is because it provides better solutions than do the more traditional approaches; specifically, this method assumes that individuals belong to different segments that are mixed in an unknown proportions and allocates consumers to the segments according to distribution probabilities (McLachlan & Basford, 1988).

## Results and discussion

### Principal component analysis (PCA): co-creation value and activities in fashion retailing

#### PCA of co-creation value (CV) in fashion retailing

As a first result in the EFA, we noticed that Kaiser–Meyer–Olkin (KMO) is > 0.8 (0.944), and Bartlett’s test was highly significant (0.0000), indicating good model acceptability and allowing us to proceed with a factor analysis of the data (Bartlett, 1954; Kaiser, 1970).

On the other hand, Cronbach’s alpha values for each factor are higher than 0.7 (Table 4). After factor extraction, an orthogonal varimax rotation was performed on factors with eigenvalues ≥ 1.0, thus allowing minimising the number of variables having high loadings on a particular factor.

**Table 4:** Internal consistency and convergent validity: co-creation value

Factor	Indicator	Loading	Robust *t*-value	Cronbach’s α	Composite reliability	Average variance extracted
Factor 1(CV) Personal interaction with the company	CV12	.918	19.121	.839	.968	.874
	CV13	.952	20.158			
	CV14	.966	20.651			
	CV15	.947	19.108			
	CV16	.889	16.883			
Factor 2(CV) Help to other people	CV23	.917	23.255	.839	.937	.820
	CV24	.946	27.343			
	CV25	.902	24.696			
	CV26	.855	20.845			
Factor 3(CV) Responsible behaviour	CV8	.843	19.321	.939	.927	.832
	CV9	.905	19.948			
	CV10	.913	19.248			
	CV11	.912	19.421			
Factor 4(CV) Company advocacy towards other people	CV20	.784	15.830	.971	.871	.726
	CV21	.953	21.449			
	CV22	.852	19.643			
Factor 5(CV) Tolerance with the company	CV27	.720	17.497	.796	.824	.810
	CV28	.866	23.225			
	CV29	.900	24.892			
Factor 6(CV) Search for information with other people	CV1	.767	19.829	.908	.768	.702
	CV2	.784	17.170			
	CV3	.838	18.164			
Factor 7(CV) Information interchange with company	CV4	.849	16.416	.948	.900	.753
	CV5	.877	17.310			
	CV6	.869	19.672			
	CV7	.868	15.343			
Factor 8(CV) Feedback with the company	CV17	.674	14.207	.869	.701	.642
	CV18	.794	15.948			
	CV19	.801	15.605			

Robust goodness-of-fit index: S-Bχ^2^ (349 df.) = 622.1034 (*p* = 0.000); NFI = 0.905; NNFI = 0.948; CFI = 0.956; RMSEA = 0.044

Eight factors resulted from the analysis of the symptomatic variance, accounting for 84.04%. The factorial structure is consistent because all the variables have a factor loading > 0.5 for the factor to which they relate (Hair et al., 1999). The names proposed for the co-creation value (CV) factors are like those used by Yi and Gong (2013): F1: personal interaction, F2: help to other people, F3: responsible behaviour, F4: business advocacy, F5: tolerance with the company, F6: search for information, F7: information interchange with the company, and F8: feedback with the company.

#### PCA of co-creation activities (CA) in fashion retailing

In the EFA, the statistics indicate good model acceptability and allow us to proceed with a factor analysis (KMO = 0.943). Moreover, the Cronbach’s alpha value is higher than 0.7 (Table 5).

**Table 5:** Discriminant validity of the theoretical construct measures: co-creation value

	F1(CV)	F2(CV)	F3(CV)	F4(CV)	F5(CV)	F6(CV)	F7(CV)	F8(CV)
F1(CV)	**.874**	[.421;.669]	[.340;.592]	[.380;.616]	[.339;.599]	[.339;.583]	[.477;.673]	[.242;.482]
F2(CV)	.297	**.820**	[.788;.912]	[.750;.886]	[.669;.841]	[.521;.741]	[.557;.341]	[.133;.381]
F3(CV)	.217	.722	**.832**	[.741;.873]	[.604;.796]	[.463;.683]	[.308;.528]	[.130;.374]
F4(CV)	.248	.669	.651	**.726**	[.628;.812]	[.449;.707]	[.265;.489]	[.117;.349]
F5(CV)	.219	.570	.490	.518	**.810**	[.653;.879]	[.421;.649]	[.304;.528]
F6(CV)	.212	.398	.328	.316	.546	**.702**	[.537;.713]	[.272;.500]
F7(CV)	.330	.201	.174	.142	.286	.390	**.753**	[.345;.561]
F8(CV)	.131	.066	.063	.054	.173	.148	.205	**.642**

The diagonal represents the AVE; above the diagonal, the 95% confidence interval for the estimated factor correlations is provided; below the diagonal, the shared variance (squared correlations) is represented

Two factors resulted from the analysis of the symptomatic variance, accounting for 66.36%, named as follows: F1: direct co-participation with the company and F2: co-participation with other people. The factorial structure is consistent because all the variables have a factor loading > 0.5 for the factor to which they relate (Hair et al., 1999).

For the behavioural response factors (engagement, satisfaction, and intention to continue co-creating), we have proceeded in a similar vein, obtaining one factor for each variable. As they are variables previously validated in various research, we do not contain the results because of spatial restrictions. The data are available from the corresponding author upon request.

Before entering the eight co-creation value factors and the two co-creation activity factors into a Latent Gold Segmentation (LGS), the content, convergent and discriminant validity and reliability of the constructs were assessed by means of confirmatory factor analysis (CFA), as we will explain next.

### Confirmatory factor analysis (CFA): co-creation value and activities in fashion retailing

#### CFA of co-creation value (CV) factors

The results of the final CFA suggest that our model provides a good fit to the data based on several fit statistics. Content validity is ensured by the use of scales that have been validated in previous co-creation studies (e.g. Constantinides et al., 2015; Nysveen & Pedersen, 2014; Yi & Gong, 2013). Cronbach’s alpha values exceeded the recommended value of 0.70, which assures the internal consistency of the measures. Convergent validity is tested by observing that all factor loadings are higher than 0.60 and significant, and the average factor loading in each factor is higher than 0.70. Additionally, average variances extracted (AVE) and composed reliability (CR) exceed, respectively, the recommended values of 0.5 and 0.7 (see Table 4).

The constructs also have discriminant validity, as none of the 95% confidence intervals for each correlation between factors included the value of 1.0, and the shared variance between each pair of constructs was always less than the corresponding AVE (Table 5). This result gives us a response to the first research objective of our investigation.

#### CFA of co-creation activity (CA) factors

Also, the results of the CFA suggest that our model provides a good fit to the data based on a few fit statistics, and reliability, as well as content, convergent and discriminant validity are assured (see Tables 6 and 7).

**Table 6:** Internal consistency and convergent validity: co-creation activities

Factor	Indicator	Loading	Robust *t*-value	Cronbach’s α	Composite reliability	Average variance extracted
Factor 1(CA) Direct co-creation with the company	CA3	.852	23.323	.920	.890	.699
	CA4	.868	23.631			
	CA6	.852	24.019			
	CA8	.774	20.687			
	CA9	.831	19.985			
Factor 2(CA) Co-creation with other people	CA1	.779	20.070	.850	.771	.589
	CA2	.842	21.769			
	CA5	.748	18.478			
	CA7	.693	18.705			

Robust goodness-of-fit index: S-Bχ^2^ (26 df.) = 50.6805 (*p* = 0.002); NFI = 0.976; NNFI = 0.983; CFI = 0.988; RMSEA = 0.049

**Table 7:** Discriminant validity of the theoretical construct measures: co-creation activities (CA)

	F1(CA)	F2(CA)
F1(CA)	**.699**	[.586;.658]
F2(CA)	.386	**.589**

The diagonal represents the AVE; above the diagonal, the 95% confidence interval for the estimated factor correlations is provided; below the diagonal, the shared variance (squared correlations) is represented

As we did with the EFA, for the behavioural response factors (engagement, satisfaction, and intention to continue co-creating), we have proceeded in a similar vein, obtaining one factor for each variable. As they are variables previously validated in numerous studies, we do not include the results due to spatial limitations. The data are available from the corresponding author upon request.

### Latent segmentation: a typology of users based on their co-creation behaviours towards the fashion retailer sector

The indicators we used to run the Latent Class Segmentation were the weighted average of each factor calculated by weighting each item by its standardised load and then adding them. Based on Constantinides et al. (2015), Del Chiappa and Lorenzo-Romero (2014), Del Chiappa et al. (2018), Frasquet-Deltoro et al. (2019) and Lorenzo and Constantinides (2019), the following indicators and covariates have been included in the segmentation model (Table 8).

**Table 8:** Measured variables: indicators and covariates

Var	Item measured	Category
I N D I C A T O R S	CO-CREATION VALUES (CV): F1: personal interaction F2: help to other people F3: responsible behaviour F4: business advocacy F5: tolerance with the company/brand F6: search for information F7: information interchange with the company/brand F8: feedback with the company/brand CO-CREATION ACTIVITIES (CA): F1: direct co-participation with the company/brand F2: co-participation with other people ENGAGEMENT with the company/brand SATISFACTION with the co-creation process INTENTION TO CONTINUE ACTIVELY CO-CREATING	Seven-point Likert scale (from strongly disagree to strongly agree)
C O V A R I A T E S	Gender	Man Woman
	Age	< 18 18–25 26–40 41–65 > 65
	Level of education	Primary school Secondary school High school Professional school University Master/postgraduate course Doctorate
	Work situation	Student Candidate for a public position Self-employed Employee Housewife Retired Unemployed
	Marital status	Single Married With partner Divorced/separated Widow/widower
	Income	Without income Far below that number Close to that number Above that number Well above that number

The first step in applying the latent segmentation method is deciding on the number of groups. The Bayesian information criterion (BIC) is used the identify the model with the least number of groups that provides the best model model fit. The best solution was the obtention of three different groups of co-creators, as the BIC was minimised in this case (Vermunt & Magidson, 2005).

**Fig. 1.**
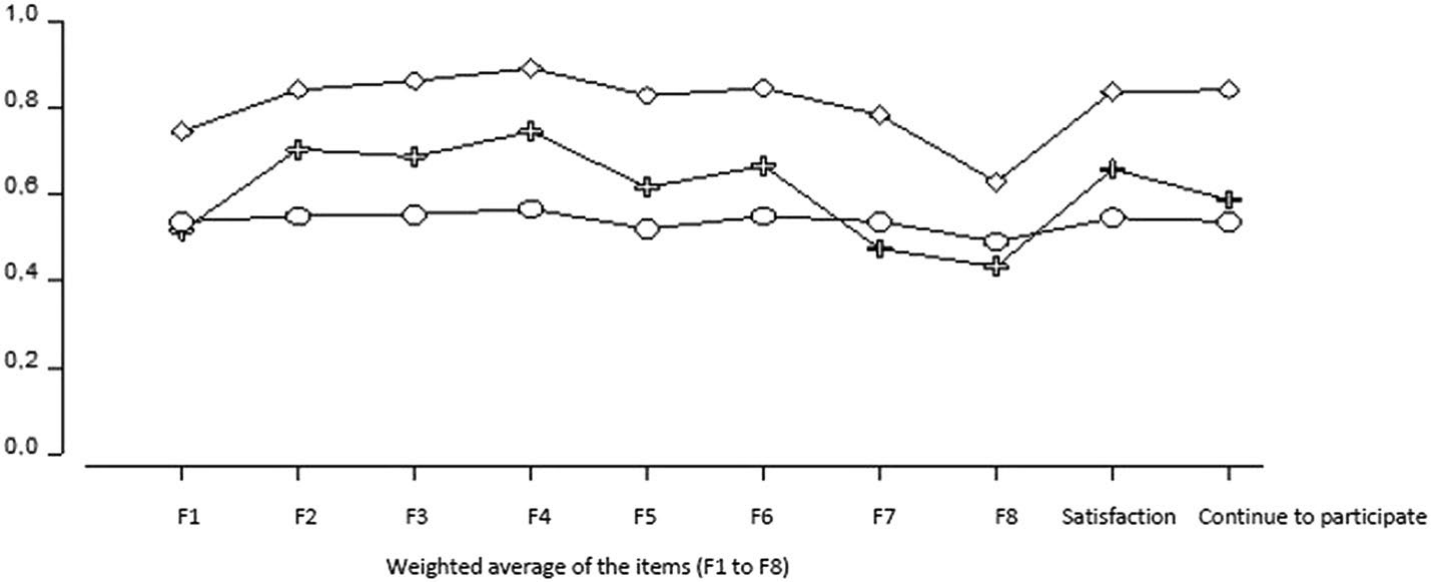
Profiles of the three clusters (average score of the indicators, and size)

Table 9 and Fig. 1 illustrate the profiles of the three clusters based on the average score of the indicators, and their size. We named the clusters according to their profile: The cluster named “co-creators oriented to the company/brand” includes 42.47% of co-innovators; the “full co-creators” segment includes 33.25% of co-innovators; and the “co-creators oriented to others” segment includes 24.29% of co-innovators.

**Table 9:** Profile of segments of co-creators (indicators)

	Co-creator oriented to the company/brand	Full co-creator	Co-creator oriented to others	Wald	*p*
Cluster size	42.47%	33.25%	24.29%		
*Indicator*					
CV: personal interaction with the company/brand	5.2890	6.2609	4.0070	234.9291	.000
CV: help to other people	3.7272	5.7884	5.2032	393.5208	.000
CV: responsible behaviour	4.9825	6.0986	4.2681	248.9486	.000
CV: business advocacy	4.8506	6.1166	4.3818	380.6148	.000
CV: tolerance with the company/brand	3.3503	4.7691	4.2691	76.6654	.000
CV: search for information with other people	3.8397	5.5197	4.9167	115.2604	.000
CV: information interchange with the company/brand	5.0656	5.9891	4.1447	237.0608	.000
CV: feedback with the company/brand	4.4963	6.0254	4.2926	581.5131	.000
CA: direct co-participation with the company/brand	2.8485	3.7973	1.7593	115.5691	.000
CA: co-participation with other people	2.4975	3.0903	3.1634	86.5268	.000
Brand engagement	5.0258	6.6401	4.0998	41.3277	.000
Co-creation satisfaction	4.6604	5.9665	4.5072	20.3746	.000
Intention to continue co-creating	4.3634	5.9572	4.5143	31.2295	.000

*CV* Co-creation value, *CA* Co-creation activities

With the purpose of refine the resulting segments and provide more information on their characteristics, we analysed different descriptive variables—or covariates—that could be related to co-creation behaviours of users (Table 10), specifically gender, age, level of education, work situation, marital status, and income. The Wald statistic indicates that significant differences (≥ 95% confidence level) exist between the segments regarding marital status, and those differences are significant at the 10% level for age, level of education, work situation, and income. Concerning gender, all clusters display a balanced participation of men and women.

**Table 10:** Profile of segments of co-creators (covariates)

Descriptive criteria (covariates)	Category	Co-creator oriented to the company/brand	Full co-creator	Co-creator oriented to others	Wald	*p*
Gender	Female	51%	48%	49%	.0865	.96
	Male	48%	51%	50%		
Age	< 18	1%	1%	1%	2.7355	.05*
	18–25	15%	16%	19%		
	26–40	34%	35%	37%		
	41–65	45%	44%	38%		
	> 65	4%	3%	4%		
Level of education	Primary school	1%	2%	1%	11.0102	.05*
	Secondary school	3%	5%	6%		
	High school	11%	11%	14%		
	Professional school	19%	23%	25%		
	University	44%	43%	43%		
	Master/postgraduate course	18%	14%	7%		
	Doctorate	1%	1%	1%		
Work situation	Student	12%	9%	13%	6.8869	.05*
	Candidate for a public position	0%	2%	2%		
	Self-employed	7%	10%	9%		
	Employee	53%	55%	56%		
	Housewife	3%	5%	3%		
	Retired	6%	6%	4%		
	Unemployed	17%	10%	11%		
Marital status	Single	35%	22%	28%	11.2109	.03*
	Married	39%	48%	44%		
	With partner	17%	24%	27%		
	Divorced/separated	2%	1%	0%		
	Widow/widower	2%	0%	0%		
Income	Without income	11%	13%	13%	6.4298	.06
	Far below that mean	23%	17%	25%		
	Close to that mean	34%	30%	30%		
	Above that mean	25%	31%	27%		
	Well above that mean	5%	8%	4%		

*Confidence level: 95%

Based on Table 9 (indicators) and Table 10 (covariates), a detailed description of each segment will be provided next, which will give us a response to the third research objective of our investigation. Firstly, the “co-creator oriented to the company/brand” segment shows a higher mean in co-creation value (CV): personal interactions with the company (5.2890) and CV: information interchange with the company (5.0656) as co-creation values. High means in CV: responsible behaviour (4.9825) and CV: business advocacy (4.8506) also show co-creation behaviours of this cluster focused on the brand. Regarding co-creation activities (CA), CA: direct co-participation with the company (2.8485), compared with CA: co-participation with other people (2.4975), also indicates, albeit with less intensity, a co-creation behaviour of this segment oriented to the company. In this segment, engagement towards the company/brand (5.0258) is higher than the satisfaction with the co-creation process (4.6604) and the intention to continue co-creating (4.3634). This cluster is the largest in the sample (42.47%) and is mainly made up of females (51%), employees (53%) between 41 and 65 years of age (45%), and those with a university degree (44%), being single and married in similar proportion (35% and 39%, respectively). In comparison with the other clusters, the “co-creator oriented to the company/brand” segment shows the highest percentage of people of a medium age (41–65 years old) and the lowest percentage of young people (18–25 years old).

Secondly, the “full co-creator” cluster has the highest scores in all co-creation behaviours (i.e. co-creation values, co-creation activities, engagement, satisfaction, and actively continuing co-creating). This segment shows the highest means (more than 6 points) in CV: personal interactions with the company (6.2609), CV: business advocacy (6.1166), and CV: feedback with the company/brand (6.0254). Related to co-creation activities for this segment, the comparison between CA: direct co-participation with the company (3.7973) and CA: co-participation with other people (3.0903) indicates that co-creators become more involved with the brand in their co-creation activities. “Full co-creators” show lower scores regarding satisfaction with the co-creation process (5.9665) and the intention to continue co-creating (5.9572) than regarding engagement with the brand (6.6401). This segment is mainly made up of married consumers (48%) between 41 and 65 years of age (44%), and those holding a university degree (43%) and employees (53%).

Thirdly, the “co-creator oriented to others” cluster shows higher mean values in CV: help to other people (5.2032) and CV: search for information with other people—remember that 1 = strongly disagree and 7 = strongly agree—with respect to co-creation values. Regarding co-creation activities, CA: direct co-participation with the company (1.7593), compared with CA: co-participation with other people (3.1634), also indicates that the co-creation behaviour of this segment is oriented to others. In this segment, as the main difference in comparison to the others, engagement with the brand (4.0998) has a lower score than does satisfaction with the co-creation process (4.5072) and the intention to continue co-creating in the future (4.5143). The profile of this cluster is composed of younger people than in the profile of other segments (37% are 26–40 years of age, and 38% are 41–65 years of age), who have university degrees (43%) and are mainly married (44%).

## Conclusions

The impact of globalisation and the growth of emerging markets are two of the variables responsible for the development that the fashion industry has experienced in recent years. The leading brands in the market have adopted a proactive attitude and incorporated emerging markets into their strategies. Brands are considering this effect and reinventing themselves in their marketing strategies to adapt to these emerging markets (González-Romo & Plaza-Romero, 2017). The trade enterprise has been driving the wave of ICT-pushed transformation with the aid of using integrating ICT into retail shops to decorate communique and interplay with clients (Kim et al., 2020).

Because of distinctive purchaser behaviours, marketplace subdivision has been broadly seemed as one of the relevant standards of marketing, “representing an effort to increase … orientation accuracy” (Kotler, 1997, p.250) via way of means of making groups awareness on the ones shoppers whom they have the finest hazard of satisfying (Dibb, 1998). Related to this idea, and according to López-Navarro and Lozano-Gómez (2014), customers should be able to choose the extent to which they participate in generating their own experiences; therefore, companies should provide a wide range of alternatives in order to meet customers’ desires (Prahalad & Ramaswamy, 2004b).

The purchaser knowhow in is worried with the quantity of inner and subjective answers to any touch with a company (Vehoef et al., 2009). From a style enterprise perspective, a complete customer revel in alludes to strategic enterprise common sense that portrays developing an advanced customer revel in alongside transactions with clients as a supply of aggressive gain for a firm (Kim et al., 2014). Therefore, as individuals and companies engage in a process of value creation together, rather than products and services, their experiences of co-creation form the new basis of value creation (Ramaswamy, 2011).

Based on previous premises, in this research we have developed a segmentation study based on some co-creation behaviours (i.e. co-creation values and activities, engagement with the brand, satisfaction with the co-creation process, and the intention to continue co-creation) in order to obtain different clusters of users while attending to their sociodemographic characteristics.

As the main differences between clusters, the “full co-creator” shows harder co-creation behaviours in all indicator variables (i.e. factors in relation to co-creation values, co-creation activities, engagement with the brand, satisfaction with the co-creation process, and the intention to continue co-creating). Moreover, this co-creator profile has a behaviour oriented towards the company with more intensity than amongst those oriented towards the other people (even higher than the scores of the “co-creator oriented to the brand” segment). However, the scores of co-creation behaviours towards other people are higher than for the “co-creator oriented to other people” cluster. Young people are more oriented towards co-creating with other people, and older people are more oriented towards co-creating with the company during their co-creation process. The “co-creators oriented to the company/brand” segment shows a lower percentage of co-creators in professional school, as the level of education, than does the “co-creator oriented to other people” cluster. On the other hand, single people are oriented towards co-creating with the company in a higher proportion than in the other two clusters. In contrast, married people are “oriented to co-create with other people” in a higher percentage than in the other groups (although they are close to the “full co-creator” segment).

In sum, in order to respond to the general research objective of our investigation, this paper analyses online co-creation behaviours in fashion retailing through a segmentation approach. Co-creation activities via social media are increasingly used by retailers to build customer engagement and loyalty, but research on this field is still limited. Our study investigated online co-creation behaviours related to a specific company/brand, combining personal characteristics to obtain different co-creator profiles. The information obtained in this research could be useful for fashion retailers to motivate different segments of users to participate actively in co-creation activities. Fashion retailers should identify the activities that different groups of customers would expect and would be the most valuable to them. Market segmentation is a strategy often used by retailers (Bevan, 2002; McGoldrick, 2002), which would suggest it is effective according to the criteria set out in the literature on marketing segmentation. It could improve the online experience in fashion retail and, as a consequence, increase sales, offering different products adapted to different obtained segments. However, it is difficult to identify all of the potential customers, which is evidenced by perennial reductions and unsold stock.

Thanks to globalisation and digitalisation, Millennials have grown. Their reality is connected to the Internet, they are less engaged with brands, and their friends’ opinions have a higher influence than that of brands. The leading brands are using influencers who are highly influential amongst Millennials, who are adapting the ways of communicating with them (González-Romo & Plaza-Romero, 2017), as a segment of special relevance in the digital environment. In this research, we can observe this fact in the obtained “co-creator oriented to others” segment, in which the highest percentage are Millennials. This segment co-creates but, compared with the other segments, they prefer activities related to co-participation with other people, and regarding co-creation value, they give more importance to helping other people and searching for information with other people. Therefore, fashion retailers should offer activities not only related to co-creating with brands, but also related to sharing content between customers, which means peer-to-peer activities. We can observe that the most brand-engaged and brand-satisfied customers are “full co-creators”. Furthermore, the “co-creator oriented to others” and “co-creator oriented to the company/brand” segments give high scores regarding these variables, and their intention to continue co-creating is also high. Therefore, these findings suggest companies to adapt their marketing programmes, particularly on social media, so as to connect with customers to improve their purchase decision process, accompanying them at each stage of the sales funnel and improving their post-purchase behaviour-engagement, satisfaction, and intention to continue co-creating, which is very important to gaining loyal customers.

Regarding academic and theoretical implications, we have demonstrated that different activities can be developed and there may be different co-creation values in the fashion retail industry, adding value to the scarce existing literature in this field (Thomas et al., 2020). In addition, demonstrating the heterogeneity of co-creator customers requires the existence of a segmentation strategy by retailers in the fashion industry to adapt to each segment and, thus, obtain greater engagement and satisfaction.

As the main limitations, we have found the following ones: difficulty in finding a valid sample fulfilling the specific requirements (fashion online co-creators) due to only 21% of Spanish fashion online buyers being co-creators with brands (INE, 2018). Moreover, there is some scarcity of literature directly related to this line of research on the segmentation in emerging markets of co-creators. Moreover, our study has analysed only one country. A possible comparison of several leading countries in the fashion industry, such as the UK and Italy, would provide more information. Therefore, this study’s finding should be taken with caution by fashion retailers in other countries, or in international organisations, although we have demonstrated that customers develop different co-creation activities, so it is interesting to allow customers to perform different co-creation activities.

As a future line of research, we propose the analysis of different obtained profiles (e.g. high versus low level of co-creation practitioners) with respect to their cooperation behaviour towards other users (family/friends versus other users) during the co-creation process and, as a consequence, the associated costly altruistic behaviour. Within this field, Ng (2020) studied the use of Facebook to analyse the degree of impact of genetic relatedness in helping others to use it, finding that passive social network users tended to provide help to those genetically related. However, active Facebook users were more willing to help social-network-known people and strangers. Both cases involved high and low biological costs related to altruistic behaviour.

## Data Availability

The datasets used and analysed during the current study are available from the first author on reasonable request.
